# Distinct classes of potassium channels fused to GPCRs as electrical signaling biosensors

**DOI:** 10.1016/j.crmeth.2021.100119

**Published:** 2021-11-22

**Authors:** M. Dolores García-Fernández, Franck C. Chatelain, Hugues Nury, Anna Moroni, Christophe J. Moreau

**Affiliations:** 1 Université Grenoble Alpes, CNRS, CEA, IBS, 71, av. Martyrs, CS10090, 38044 Grenoble Cedex9, France; 2 Université Côte d’Azur, IPMC CNRS UMR7275, Laboratory of Excellence ICST, 660 route des Lucioles, 06650 Valbonne, France; 3 University of Milan, Department of Biosciences, Via Celoria 26, 20133 Milano, Italy

**Keywords:** man-made ligand-gated ion channels, biosensors, protein engineering, electrical signal, ion channels, K2P channels, viral channels, G protein-coupled receptors, allosteric regulation

## Abstract

Ligand-gated ion channels (LGICs) are natural biosensors generating electrical signals in response to the binding of specific ligands. Creating *de novo* LGICs for biosensing applications is technically challenging. We have previously designed modified LGICs by linking G protein-coupled receptors (GPCRs) to the Kir6.2 channel. In this article, we extrapolate these design concepts to other channels with different structures and oligomeric states, namely a tetrameric viral Kcv channel and the dimeric mouse TREK-1 channel. After precise engineering of the linker regions, the two ion channels were successfully regulated by a GPCR fused to their N-terminal domain. Two-electrode voltage-clamp recordings showed that Kcv and mTREK-1 fusions were inhibited and activated by GPCR agonists, respectively, and antagonists abolished both effects. Thus, dissimilar ion channels can be allosterically regulated through their N-terminal domains, suggesting that this is a generalizable approach for ion channel engineering.

## Highlights

• GPCR regulation of diverse potassium channels through N-terminal fusions
• Fusion to the viral PBCV-1 Kcv channel requires a specific linker and receptor
• Tetrameric Kcv and dimeric mTREK-1 channels fusions respond to GPCR ligands
• The engineered fusions broaden the properties of the channels' electrical signals

## Motivation

For biosensing applications, electrical signals are attractive since they can be recorded by micro- or nano-electronic systems for developing miniaturized detection devices in biomedical or environmental fields. One of the main limiting steps is the design of sensing elements that specifically recognize ligands such as biomarkers or chemical compounds. Ligand-gated ion channels are natural biosensors that specifically recognize ligands and generate an electrical signal. They have naturally evolved to be finely tuned by regulatory domains or proteins resulting in an appropriate electrical signal. The objective of this work is to leverage these natural biosensors by engineering diverse artificial ion channels that have the desired properties for biosensing or basic research applications. The main challenge is to design *de novo* allosteric regulations between ion channels and physiologically unrelated membrane proteins. Previously, different G protein-coupled receptors were successfully coupled to a specific ion channel, Kir6.2. In this work, we demonstrate that other ion channels (viral and murine) with distinct oligomerization properties can also be fused to GPCRs via their N-terminal domains. These results open avenues for diversifying engineered ligand-gated ion channels.

## Graphical abstract

**Figure:**
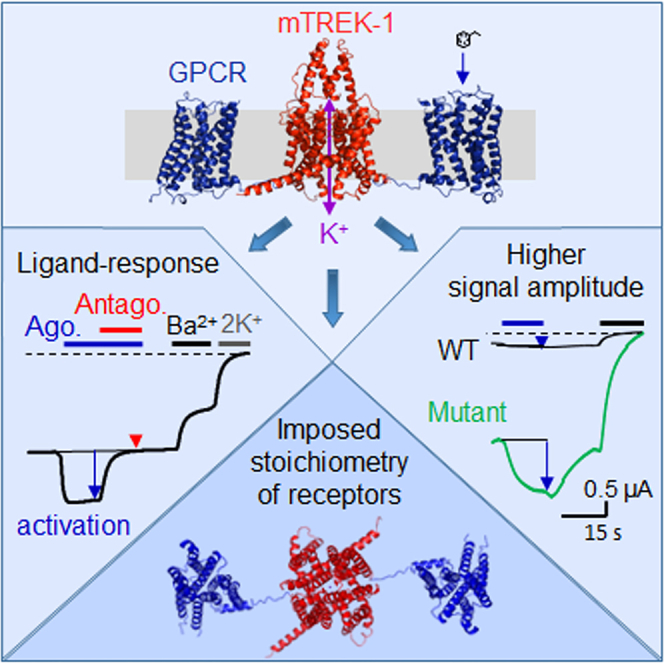

## Teaser

Ligand-gated ion channels are natural biosensors that generate highly sensitive and fast electrical signals. García-Fernández et al. broaden the regulatory properties of electrical signal output by fusing different potassium channels to GPCRs. Functional fusion to tetrameric and dimeric channels demonstrates the versatility of this approach.

## Introduction

Ligand-gated ion channels (LGICs) are natural biosensors that transform chemical stimuli into electrical signals. These signals can be recorded by standard electrophysiological techniques or micro- and nano-electronic systems. LGICs have some limitations that restrain applications in biosensing, such as the low number of recognized ligands and the short-lived signal induced by intrinsic inactivation process. A new class of man-made LGICs has been designed by physical and functional coupling of different G protein-coupled receptors (GPCRs) to an ion channel (Moreau et al., 2008). The GPCR moiety plays the role of the ligand-binding domain while the fused ion channel forms the ion-selective pore. These man-made LGICs, called ion channel-coupled receptors (ICCRs) broaden the properties of LGICs as: (1) they diversify the range of ligands to those recognized by the fused GPCRs, (2) they generate a sustainable signal, (3) they are selective to potassium, and (4) they have a basal activity that preserves a negative membrane resting potential and allows the detection of both activating and inhibiting ligands. The ICCR technology has multiple applications both in applied research as biosensors in interface with nano-electronic systems (Lim et al., 2015) and in basic research for functional studies of ion channels (Moreau et al., 2017; Principalli et al., 2017) and GPCRs (Moreau et al., 2015; Niescierowicz et al., 2014).

The ICCRs were initially conceived as versatile tools with simple protein engineering for swapping the fused GPCR with another one. Several ICCRs were successfully designed with the following GPCRs: human M2 (M2) (Moreau et al., 2008), human D2_L_ (D2) (Moreau et al., 2008), human β2 adrenergic (Caro et al., 2011), bovine rhodopsin (Caro et al., 2012), human OR2AG1 olfactory (Lim et al., 2015), and human oxytocin (OXTR) (Niescierowicz et al., 2014) receptors. ICCRs are created by fusing the GPCR C termini to the N terminus of the Kir6.2 ion channel. Additional engineering steps are required to create a functional coupling between the two proteins in order to regulate the ion channel gating by the conformational changes of the fused GPCR. The length of the linking region must be adjusted (Moreau et al., 2008) by deleting the first 25 residues of Kir6.2 (Moreau et al., 2017) and by adjusting the length of GPCR C termini to that of the M2 or D2 receptor. This empirical approach appeared to be partially applicable to some GPCRs but not to all of them for reasons that are still unknown. We postulate in this work that changing the ion channel part could facilitate the design of ICCRs and broaden the properties of the signal. Kir6.2 was initially selected because it is naturally regulated by large transmembrane proteins, the sulfonylurea receptors (SURs) (Inagaki et al., 1995; Martin et al., 2017; Lee et al., 2017; Ding et al., 2019). While the molecular mechanism of this allosteric regulation is not precisely defined, structural evidence suggests that multiple domain-domain interactions are involved in the propagation of conformational changes from SUR ligand-binding sites to Kir6.2 gates (Martin et al., 2019). Similarly, in ICCRs, both the N- and C-terminal domains of Kir6.2 play a role in the regulation by the fused GPCR (Principalli et al., 2017).

While Kir6.2 naturally evolved to be allosterically regulated by large transmembrane proteins, this is an exception in the ion channel superfamily, and we asked whether dissimilar ion channels can be also regulated through N-terminal fusions. Ion channels devoid of interactions between domains could simplify the engineering of ICCRs and facilitate the development of a standard and rational protocol for their design. To explore this hypothesis, two potassium channels were chosen from different families and with different stoichiometries.

First, the viral Kcv channel from *Paramecium bursaria* chlorella virus 1 (PBCV-1) (Plugge et al., 2000; Romani et al., 2013) was selected. For the sake of simplicity, PBCV-1 Kcv is denoted Kcv in this article. Kcv is one of the smallest known potassium channel (94 residues versus 390 for Kir6.2) containing only a short cytoplasmic N-terminal domain (12 residues) and no cytoplasmic C-terminal domain, according to predicted models (Hoffgaard et al., 2015; Andersson et al., 2018). This channel is tetrameric like Kir6.2, and it has already been successfully engineered to create artificial voltage- and light-gated ion channels (Arrigoni et al., 2013; Cosentino et al., 2015; Alberio et al., 2018).

**Figure 1:**
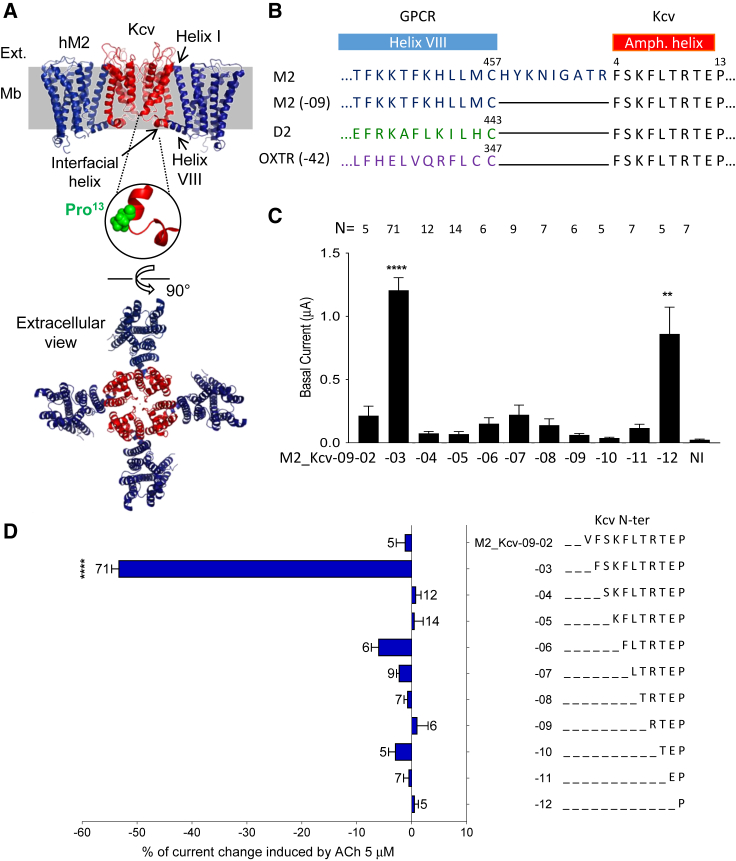
M2_Kcv ICCR is ligand regulated with precise linker length (A) Diagram of hM2_Kcv-09-00 based on the structure of the human muscarinic M2 (hM2) receptor (Maeda et al., 2019) (PDB: 6OIK) and the model of Kcv (Hoffgaard et al., 2015). The left figure shows a view from the membrane plane (Mb, in grey) with M2 receptors in blue and the tetrameric Kcv in red. Front and back M2 receptors are masked for clarity. The amphipathic Kcv interfacial helix and the M2 helix VIII are indicated. The M2 helix I is also designated. A zoom on the Kcv Proline 13 (Pro^13^, in green) is shown under the figure. Ext., extracellular. The lower figure shows an extracellular view of the full complex (4 M2 and 4 Kcv). The pore is in the middle of the complex with a potassium ion shown as a dot. The position of M2 relative to the pore is determined by the extension of the Kcv interfacial helix with the M2 helix VIII. (B) Alignment of the linking region showing the full-length M2 C terminus (M2), the M2 C terminus truncated of its last nine residues (M2 (−9)), the D2_L_ C terminus (D2), OXTR C terminus truncated of its last 42 residues (OXTR (−42)) and the Kcv N terminus truncated of its first three residues. The nomenclature of ICCRs are GPCR-channel-x-y with x residues deleted at the GPCR C terminus and y residues deleted at the channel N terminus. Positions of the GPCR helix VIII (blue) and the putative Kcv amphipathic helix (red) are represented above the sequences. (C) Amplitude of the basal currents recorded by TEVC on *Xenopus* oocytes expressing the indicated M2_Kcv constructs. N is indicated over bars. NI, non-injected oocytes. Kruskal Wallis test post Dunn's test; ∗∗p < 0.01, ∗∗∗∗p < 0.0001 (ref.: NI). (D) Percentage of current change induced by 5 μM ACh on the indicated ICCRs expressed in *Xenopus* oocytes. TEVC recordings were performed in external 91 mM K^+^ at Vm = −50 mV. In M2_Kcv, the last nine residues of M2 are deleted (−09) and incremental deletions in Kcv N-terminal domain were performed (−02 to −12). Negative values indicate an inhibition of the fused Kcv. One sample t test; ∗∗∗∗p < 0.0001. Number of recordings (N) is indicated beside the bars. Error bars are SEM.

The second chosen ion channel was the mouse Twik-related K^+^ channel 1 (mTREK-1 or mK_2P_2.1) (Fink et al., 1996). It belongs to the family of the two-pore-domain potassium channels (K_2P_) (Gada and Plant, 2019; Enyedi and Czirják, 2010), which has the particularity of functioning as dimers while most potassium channels are tetramers. Moreover, in the structure (Lolicato et al., 2017) of mouse TREK-1 (PDB: 6CQ6), the N-terminal domain is a cytoplasmic extension of the transmembrane helix 1 (TM1) pointing toward the cell interior and does not contain the interfacial helix found in Kir channels (Meng et al., 2016; Nishida et al., 2007) and predicted in Kcv. The interfacial helices are amphipathic α helices parallel to the membrane plane and anchored to the inner leaflet of the membrane by hydrophobic residues (Figure 1A). Consequently, fusion of GPCR's C terminus to mTREK-1 could directly link the receptors to the first transmembrane helix of the channel. Such a configuration has two advantages: (1) an absence of N-terminal cytoplasmic domain and interfacial helix, which should simplify the strategy of design; and (2) an imposed stoichiometry of two GPCRs per pore in the GPCR-mTREK-1 complex.

The challenge in the design of this new type of ICCR is to create *de novo* allosteric regulations of ion channels with different structures, oligomeric states, and gating.

With regard to the large diversity of ion channels, the ability to fuse different ion channels with GPCRs would open new perspectives to adjust the properties of ICCRs with desired characteristics such as higher signal amplitude, specific regulations (chemical, mechanical, or physical), and different oligomeric states. These man-made LGICs are also novel tools to study the inherent molecular mechanisms of gating through intermolecular allosteric regulation.

## Results

### M2 receptor regulates the fused Kcv channel

The first attempts to change the ion channel moiety in ICCRs were made with the Kcv channel. This channel is an attractive candidate since it has a predicted short amphipathic N-terminal helix (interfacial helix) that can be directly connected to the C-terminal amphipathic helix VIII of GPCRs (Figure 1A). The fusion of the receptor and the channel amphipathic helices could simplify the ICCR design by creating a single helix with an optimal length.

With this objective, we deleted the C-terminal nine residues of M2 (M2_Kcv-09-xx) to reach the last residue of the helix VIII, the palmitoylated Cys^457^ (Figure 1B). Then, incremental deletions of the Kcv N-terminal domain were made (Figure 1C), linking to the receptor the residues from Val^3^ to Pro^13^, the latter being at the cytoplasmic end of TM1 (Figure 1A).

The ICCRs were expressed in *Xenopus* oocytes and their expression and regulation were assessed by the two-electrode voltage-clamp (TEVC) technique. These results (Figure 1C) clearly show that two constructs (M2_Kcv-09-03 and M2_Kcv-09-12) significantly generate larger basal currents (1.21 ± 0.10 μA and 0.86 ± 0.21 μA, respectively) compared with non-injected (NI) oocytes (0.024 ± 0.005 μA). Those large currents suggest that the corresponding constructs shift the equilibrium of Kcv toward an open state. The profile of basal currents (generated in absence of ligand) suggests periodicity of about four residues, with bigger amplitudes peaking at deletions −03, −07, and −12 (Figure 1C). Such a periodicity is in line with our initial hypothesis of creating a single helix by connecting the helix VIII of the GPCR to the putative amphipathic helix of Kcv.

**Figure 2:**
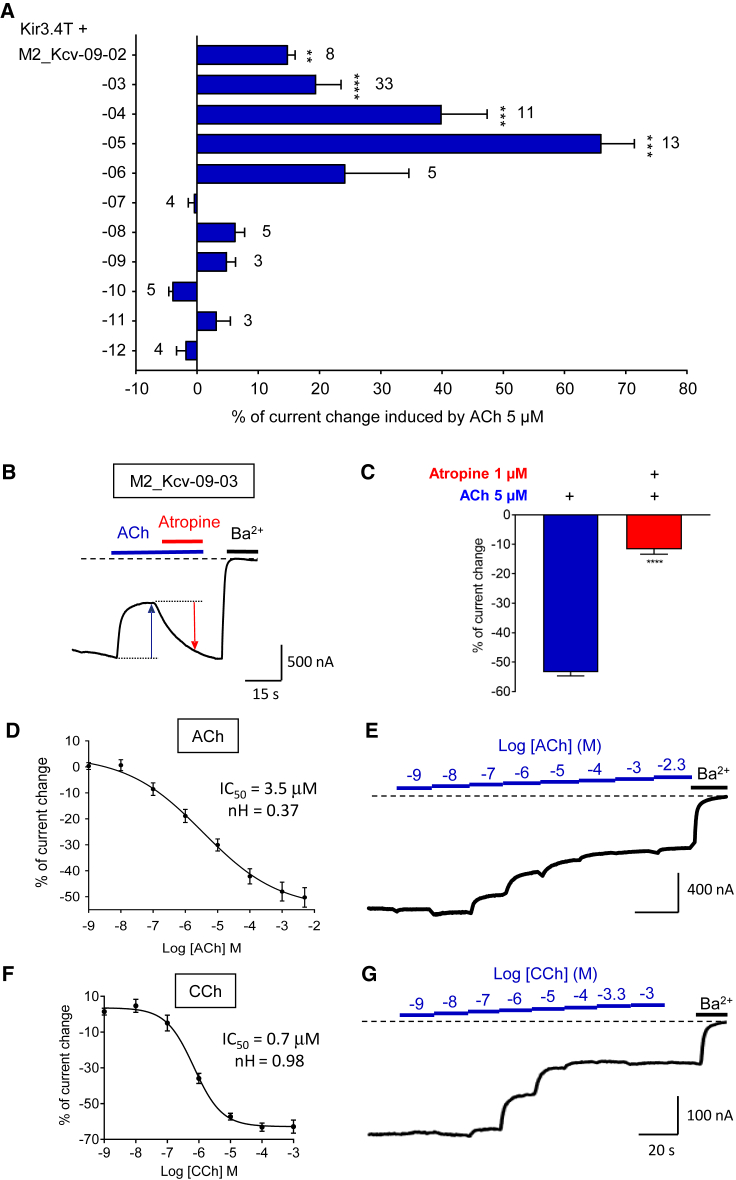
Functional characterization of M2_Kcv ICCRs (A) Percentage of current change induced by ACh 5 μM on Kir3.4T (Kir3.4(S143T) mutant) co-expressed with the indicated constructs. Positive values relates to an activation of Kir3.4T mediated by endogenous Gi/o proteins activated by the fused M2 receptor. N is indicated beside bars. Wilcoxon signed-rank test; ∗∗p < 0.01, ∗∗∗p < 0.001, and ∗∗∗∗p < 0.0001. (B) Representative TEVC recording of the functional M2_Kcv-09-03 ICCR. The dashed line represents 0 potassium current (barium sensitive). During 5 μM ACh application (blue bar), the negative current amplitude decreases (inhibition, blue arrow). Co-application of 1 μM atropine (red line) with 5 μM ACh inversed the ACh-induced inhibition (red arrow). Application of 3 mM barium (Ba^2+^), a potassium channel blocker, is illustrated by a black line. (C) Statistics of ACh and atropine responses on M2_Kcv-09-03. Mann-Whitney test; ∗∗∗∗p < 0.0001. N is 71 and 11, respectively. (D) Concentration-effect curve of ACh on M2_Kcv-09-03. N is between 5 and 35. (E) Representative TEVC recording of the results presented in (D) and showing application of incremental concentrations of ACh. (F) Concentration-effect curve of CCh on M2_Kcv-09-03. N is 13. (G) Representative TEVC recording of (F) with incremental concentrations of carbachol (CCh). In all panels, error bars are SEM.

The functional characterization of the ICCRs in presence of the M2 agonist acetylcholine (ACh) at 5 μM (Figure 1D) reveals that only M2_Kcv-09-03 is clearly functional with an inhibition of 53.4% ± 1.2%. Constructs with larger deletions up to 12 residues did not yield significant responses, which indicates that most of the Kcv N-terminal domain must be conserved to be functionally connected to the M2 helix VIII. Two reasons could explain the lack of response of the other constructs: (1) a loss of receptor activity, or (2) a lack of functional coupling between the fused proteins. To test these hypotheses, all M2_Kcv constructs were co-expressed with the homomeric G protein-activated Kir3.4(S143T) channel (Kir3.4T). Kir3.4T channels are activated by the Gβγ subunits of Gi/o proteins, which are endogenously expressed in *Xenopus* oocytes (Hatcher-Solis et al., 2014), and they thus act as reporters of GPCR activation. The results (Figure 2A) show that the deletions −02 to −06 of the Kcv N terminus did not affect the G protein activation by M2, while larger deletions impaired this function. Consequently, the lack of Kcv regulation is not related to a lack of receptor function for the constructs −02 to −06. By contrast, larger deletions have a clear detrimental effect on the receptor activity, which precludes any functional coupling with the fused channel. The lack of regulation of Kcv in constructs with receptors having the ability to activate G proteins suggests that the activation of G protein pathways is not a mechanism involved in the regulation of Kcv ICCR. To confirm this assumption, controls were made by co-expression of unfused M2 and Kcv and by expression of Kcv alone, and the results (Figures S1A–S1C) demonstrate an absence of regulation of unfused Kcv by M2 and by ACh. Consequently, the regulation of M2_Kcv-09-03 by ACh is mediated by the fusion between the receptor and the channel.

Kcv has well-characterized current-voltage relationships (Gazzarrini et al., 2002). Since our experiments are performed in voltage-clamp conditions, voltage dependence does not affect the regulation of the channels by the fused GPCRs in our experimental conditions. However, the fusion with M2 could have affected the voltage dependence of the fused channels and this could be of interest for basic research on the voltage dependence of Kcv or for applications relying on voltage modulation. Measurements of steady-state currents correlated with incremental clamped voltages (I/V curves in Figures S1D–S1F) suggest that the current-voltage relationship has been modified by the fusion of Kcv with M2, since the I/V curve is more linear than the curve with Kcv alone. Consequently, the fusion seems to affect the voltage dependence of Kcv, which must be taken into account in voltage-dependent applications of Kcv ICCRs.

The design of only one functional M2_Kcv ICCR out of 11 tested constructs highlights the high precision required in protein engineering for designing a Kcv ICCR regulated by a M2 agonist. This regulation is an inhibition of the channel, which was also observed with the equivalent Kir6.2 construct: M2_Kir6.2-09-25 (Moreau et al., 2017).

Kcv and Kir6.2 ICCRs have similar characteristics. Antagonist sensitivity, previously observed with Kir6.2 ICCRs, is preserved with Kcv as shown in Figures 2B and 2C. Co-application of 1 μM atropine with ACh 5 μM on M2_Kcv-09-03 almost entirely abolished the ACh response. Consequently, the fused Kcv channel is reversibly regulated by the agonist and the antagonist-bound states of M2. Concentration-effect curves (Figures 2D–2G) with ACh and the synthetic agonist carbachol (CCh), indicate half maximal inhibitory concentrations (IC_50_s) of 3.5 μM and 0.7 μM, respectively, which is in the same order of magnitude as IC_50_s of ACh (1.25 μM) and CCh (1.2 μM) observed with Kir6.2 ICCRs (Moreau et al., 2017). The Hill coefficients for ACh (0.37) and CCh (0.98) suggest an absence of cooperativity as observed with unfused M2 receptor (Jakubík et al., 1997).

These findings demonstrate that Kir6.2 is not the only ion channel that can be functionally coupled to a GPCR. A minimalist potassium channel, composed only of a short amphipathic helix and two transmembrane helices, can be engineered for GPCR regulation and it improves the ICCR technology with a higher signal amplitude (53.4% ± 1.2% of inhibition) compared with Kir6.2 (27% ± 3%). However, the functional coupling requires a highly precise linker engineering since only one functional construct was obtained over 11 tested deletions.

### The Kcv channel is not regulated by D2 and OXTR receptors

On the basis of the functional M2_Kcv-09-03 construct, we designed new Kcv ICCRs with other GPCRs. The human dopaminergic D2 receptor was chosen since it has already been successfully coupled to Kir6.2 (Moreau et al., 2008). The C terminus of D2 being nine residues shorter than that of M2 (Figure 1B), the fusion D2_Kcv-00-03 features the same linker size as M2_Kcv-09-03. TEVC recordings of D2_Kcv-00-03 showed neither significant amplitudes of basal currents (0.026 ± 0.005 μA) (Figure S1G) nor any regulation by the newly fused receptor (Figure S1H). To assess whether the functionality of the D2 receptor moiety persisted within the ICCR, the G protein-activated Kir3.4T channel (Moreau et al., 2015) was co-expressed with D2_Kcv-00-03. The modulation of current upon dopamine application (Figure S1I) demonstrates that D2 receptor still binds dopamine and triggers the activation of the endogenous Gi/o proteins. Consequently, the lack of ICCR response is not due to a loss of D2 receptor function.

We next engineered fusions of Kcv with another GPCR: the human oxytocin receptor (OXTR). OXTR also created a functional ICCR with Kir6.2 (Niescierowicz et al., 2014). Compared with the wild-type (WT) OXTR, the fused receptor had two modifications: (1) its third intracellular loop was replaced by the T4 phage lysozyme (T4L) domain (Cherezov et al., 2007) for crystallographic studies, and (2) its last 42 residues were truncated to match the M2 (−09) C terminus length (Figure 1B). That fused construct (OXTR[T4L]_Kcv-42-03) generated significant basal currents (0.47 ± 0.10 μA) (Figure S1G), reminiscent of the M2_Kcv-09-03 construct. However, applications of oxytocin did not induce any response (Figure S1J). To determine whether the lack of G protein activation by OXTR[T4L] was involved in the lack of ICCR regulation, a construct with intact third intracellular loop and without T4L domain was designed: OXTR_Kcv-42-25. The results in Figures S1G and S1K show that, while T4L removal decreases the basal activity of OXTR ICCR, it does not induce Kcv regulation, indicating that G protein pathways are not involved in the regulation of the Kcv ICCR.

Consequently, the linker size determined for M2 receptor cannot be directly transferred to two other GPCRs, indicating that the precise design of the functional M2_Kcv specifically suits the sequence and the conformations of the M2 receptor. It is likely that D2, OXTR, and other GPCRs could be functionally coupled to Kcv. However, the identification of the correct linker for each receptor would be fastidious and does not meet the objective of simplifying the design of ICCR. We considered that the most suitable strategy for designing fusions of Kcv with different GPCRs would be based on computational studies. However, to be absolutely accurate, this strategy requires knowledge of the three-dimensional structure of Kcv, which has not been obtained yet. In consequence, we decided to focus our efforts on the creation of fusions with another ion channel, mTREK-1.

### The homodimeric mTREK-1 channel is regulated by the M2 receptor

As it was initially the case for the Kcv channel, there is no prior evidence that TREK-1 can be allosterically regulated by a receptor through an N-terminal fusion. However, using TREK-1 as the channel moiety would provide desirable properties to ICCRs. First, the higher conductance of TREK-1 (14.8 ± 3.3 μS in K^+^-rich solution [Fink et al., 1996] versus 76.4 ± 1.0 pS for Kir6.2 [Inagaki et al., 1995]) increases the signal amplitude and would allow the detection of weakly expressed ICCRs. Second, as TREK-1 is a dimer and not a tetramer, a new stoichiometry of two GPCRs per pore is imposed. The first seen N-terminal residues of the mTREK-1 dimer are located on opposite sides of the channel. Thus, the fused GPCRs would not have direct contact with each other (Figures S2A and S2B). Third, the absence of an N-terminal amphipathic helix in the structure of TREK channels, the murine channel (mTREK-1) (Lolicato et al., 2017), and the human TREK-2 channel (Dong et al., 2015) may lead to simplified protein engineering. In absence of identified N-terminal amphipathic helix in mTREK-1, the previous optimization of Kir6.2 and Kcv N termini could not be re-exploited and the strategy of fusion between GPCRs and mTREK-1 had to be re-investigated. The M2 receptor was once again used as a GPCR archetype and we tested both a full-length and a C-terminal nine-residue truncated construct. Postulating that close proximity of the receptor to the pore would be the most efficient position to create a regulation of the channel, we linked the receptor C terminus to the proximal region of the first transmembrane helix (TM1) of mTREK-1.

In the mTREK-1 structures (Lolicato et al., 2017; Pope et al., 2020) (PDB: 6CQ6 and 6V36), the residue Ser^37^ is located at the cytoplasmic end of TM1 and points away from the pore (Figure S2C). Ser^37^ thus seemed a favorable initial position for fusing the M2 receptor without disrupting the channel function. Deletion of Δ1-66 in TREK-2 (equivalent to Δ1-41 in mTREK-1) produced functional channel with higher unitary conductance (Simkin et al., 2008). Consequently, the first mTREK-1 ICCR was M2_T-00-37 with zero deletion in the M2 receptor C terminus (full-length receptor) and 37 residues deleted in the mTREK-1 N terminus. Three additional, slightly larger, deletions in the mTREK-1 N terminus were also created, −40, −43, and later −46 (Figure S3A), with the objective of bringing the receptor even closer to the pore. The periodicity of three residues in the truncations was used to preserve the orientation of the fused residues in the TM1 α helix. The first TREK-1 residues of the deletions −37, −40, −43, and −46 (Ala^38^, Val^41^, Trp^44^, and Val^47^ respectively) belong to or surround a conserved region in the TREK-1 family. Unfused N-terminally truncated (−37, −40, and −43) mTREK-1 were initially created as controls. Deletion of the last nine residues of M2 (M2_T-09-43) was also explored in order to directly connect the M2 helix VIII with the mTREK-1 helix TM1.

**Figure 3:**
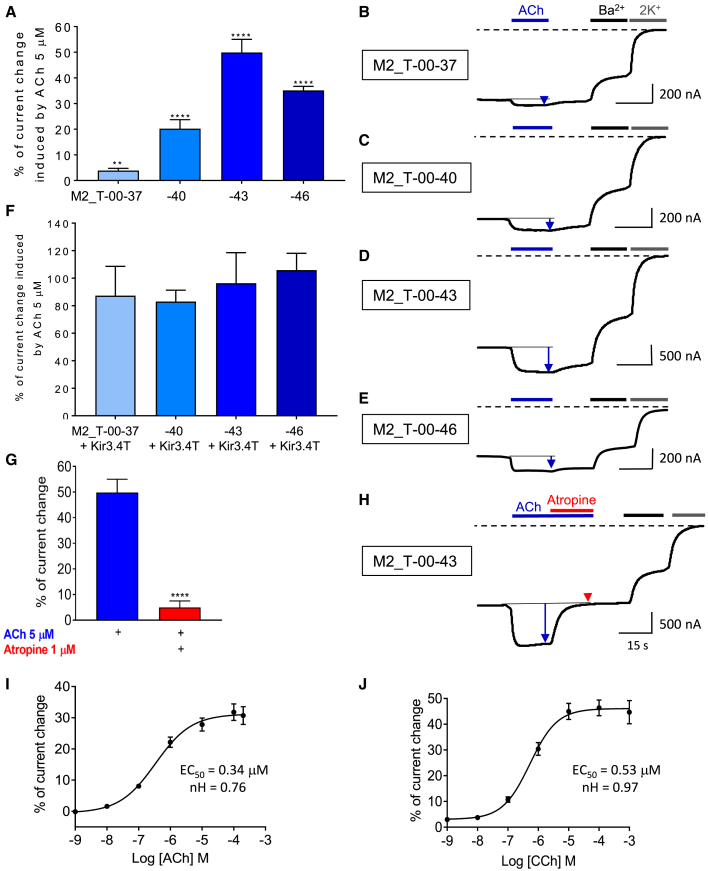
hM2_mTREK-1 ICCR is ligand regulated with different linker length (A) Percentage of current change induced by 5 μM ACh on the indicated M2_TREK-1 (M2_T) ICCRs. Positive values indicate an activation of the ICCR. The number of recordings is 12, 40, 55, and 22 for the constructs −37, −40, −43, and −46, respectively. One sample t test, ∗∗p = 0.0014, ∗∗∗∗p < 0.0001. (B) Representative TEVC recordings of M2_T-00-37 that were performed by TEVC as described in Figure 1B with addition of application of low-potassium buffer (2K^+^) in grey. (C) Representative TEVC recordings of M2_T-00-40. (D) Representative TEVC recordings of M2_T-00-43. (E) Representative TEVC recordings of M2_T-00-46. (F) Percentage of current change induced by 5 μM ACh on the G protein-activated potassium channel Kir3.4T co-expressed with the indicated M2_T ICCRs. The number of recordings is six, 36, seven, and eight for −37, −40, −43, and −46, respectively. (G) Percentage of current change induced by 5 μM ACh (blue bar) and by 5 μM ACh + 1 μM atropine (red bar) on M2_T-00-43. The number of recordings is 55 and eight for ACh and ACh + atropine applications, respectively. Mann-Whitney test. (H) Representative TEVC recordings of the results in (G). (I) Concentration-effect curve of ACh on M2_T-00-43. N is between 12 and 18. (J) Concentration-effect curve of CCh on M2_T-00-43. N is 23. Error bars are SEM. ∗∗p < 0.01, ∗∗∗∗p < 0.0001.

These constructs were heterologously expressed in *Xenopus* oocytes and functionally characterized with the TEVC method. Controls with N-terminal deletions of unfused mTREK-1 (Figures S3B–S3E) showed a similar profile of I/V curves of the truncated channels to that of the WT channel, with lower current amplitudes for all truncated channels and a lower activation by 10 μM BL1249 for mTREK-1 −43. These results show that incremental N-terminal deletions of mTREK-1 preserve the voltage-dependence characteristics and ligand sensitivity of the channel, but with lower current amplitudes correlated with the length of deletions. In ICCRs, the results (Figures 3A–3E) showed an inverted effect with an increase of the amplitude of ACh response correlated with an increase of the length of deletions from −37 to −43. Thus, a slight activation of M2_T-00-37 by ACh 5 μM (3.9% ± 0.9%) was observed, which increased with larger deletions -00-40 and -00-43 (20.2% ± 3.5% and 49.9% ± 5.1%, respectively) but decreased for the deletion -00-46 (35.2% ± 1.6%). Thus, while the regulation of the gating is decreased in unfused truncated channels when N-terminal deletions increase, shorter distances between the receptor and the channel improve the regulation of the gating in a certain range with an optimum of 43 residues deleted over the four deletions tested. This is consistent with a mechanical transduction of GPCR conformational changes to the channel gate(s), which is improved by closer contact between the two proteins until steric hindrances occur. However, another possibility is a differential effect of the different deletions on the receptor activity. To evaluate this hypothesis, the activation of G proteins by the fused receptor was assessed for each construct. The fusions M2_T-00-37, −40, −43, and −46 were co-expressed with the G protein-activated potassium channel Kir3.4T. The results (Figures 3F and S4A–S4D) show clear activation of Kir3.4T channels induced by ACh and no significant differences of the amplitudes between the four constructs. Thus, the different deletions did not differentially affect the activation of the fused receptor.

The effect of fusion on the voltage dependence of mTREK-1 was also assessed for potential applications based on voltage modulation. The I/V curves of mTREK-1 and M2_T-00-43 (Figures S4E–S4G) show a similar profile between the fused and unfused channels. The outward rectification is preserved but with a lower current amplitude for the ICCR, which was previously observed with all other fused channels.

As for the Kcv ICCR, atropine was used to test the sensitivity of M2_T-00-43 to antagonists. Co-application of 1 μM atropine to 5 μM ACh inhibited ACh-induced activation (Figures 3G and 3H). The gating of mTREK-1 is thus sensitive to both agonist- and antagonist-bound states of the M2 receptor.

Concentration-effect curves (Figures 3I and 3J) with ACh and CCh confirm the correlation between agonist concentration and signal amplitude. As observed with the Kcv ICCR, the values of EC_50_ and the Hill coefficient (nH) for CCh are in the same order of magnitude as previously observed. For ACh, the EC_50_ is decreased (0.34 μM versus 1.25 and 3.5 μM for Kir6.2 and Kcv ICCRs, respectively), while the Hill coefficient (0.76 versus 0.37 for Kcv ICCR) increases but remains lower than 1, indicating an absence of cooperativity. This higher apparent affinity for ACh with M2_T ICCR could be related to the application of high sodium buffer (2K^+^ buffer) before measuring ligand response. Friedman et al. (2020) demonstrated that removal of Na^+^ decreases the potency of ACh on the M2 receptor. High sodium buffer has been specifically used as reference before and after ligand applications with mTREK-1 ICCRs for the baseline determination since this channel is only partially blocked by barium. Kir6.2 and Kcv being efficiently blocked by extracellular barium, the barium-sensitive currents were used as reference in recordings of ICCRs composed of these channels.

**Figure 4:**
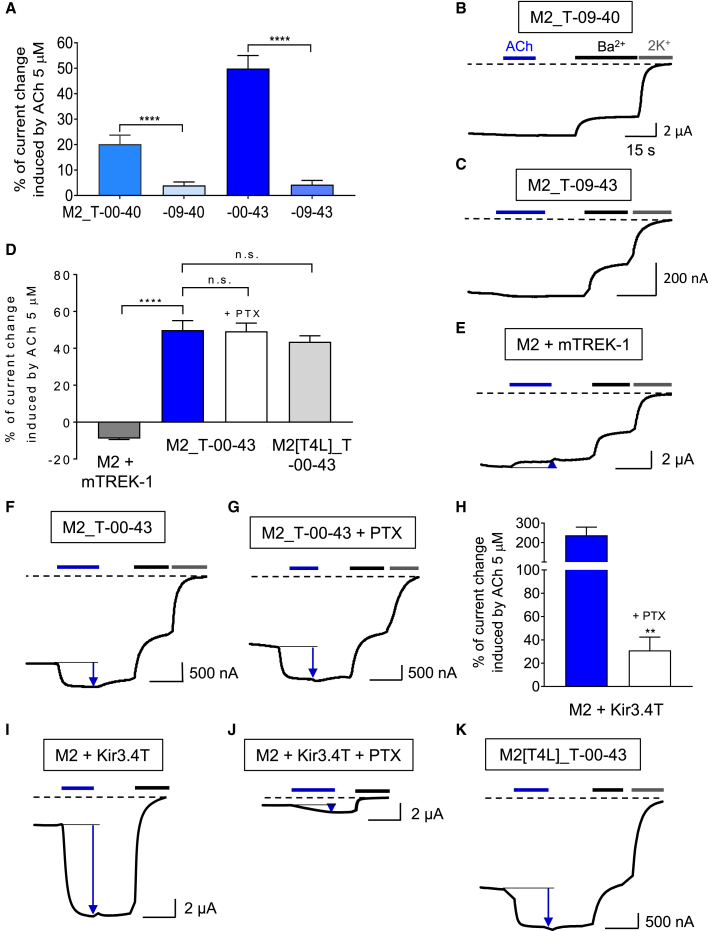
mTREK-1 ICCR is regulated through the fusion and not by intracellular effectors (A) Percentage of current change induced by 5 μM ACh on full-length (−00) or M2 deleted of its last nine residues (−09) in the indicated ICCRs. The number of recordings is 40, 17, 55, and seven for M2_T-00-40, -09-40, -00-43, and −09-43, respectively. (B and C) Representative TEVC recordings of the results in (A). (D) Percentage of current change induced by 5 μM ACh on the unfused M2 and mTREK-1 (M2 + mTREK-1, dark grey bar), the fused M2_T-00-43 without (blue bar), with co-expression of the S1 subunit of the pertussis toxin (PTX) (white bar) and the M2[T4L]_T-00-43 construct (light grey bar). The number of recordings is five, 55, 10, and 17 for the constructs M2 + mTREK-1, M2_T-00-43 without and with PTX, and M2[T4L]_T-00-43, respectively. n.s., not significant, p = 0.11 and p = 0.31 for M2_T-00-43 + PTX and M2[T4L]_T-00-43, respectively. (E) Representative TEVC recordings of M2 co-expressed with unfused mTREK-1. (F) Representative TEVC recordings of M2_T-00-43. (G) Representative TEVC recordings of M2_T-00-43 co-expressed with PTX. (H) Percentage of current change induced by 5 μM ACh on the unfused M2 and the G protein-activated Kir3.4T. Control experiment showing the inhibition of Gi/o activation by the co-expressed PTX. The number of recordings is six and four for the conditions in absence and in presence of PTX, respectively. ∗∗p = 0.0095. (I and J) Representative TEVC recordings of the results in (H). (K) Representative TEVC recording of the results of M2[T4L]_T-00-43 in (D). In all panels, recordings were performed by TEVC as described in Figure 1B. Mann-Whitney test, ∗∗∗∗p < 0.0001. Error bars are SEM.

In a second round of design, ICCRs bearing a deletion of the last nine residues from the M2 receptor moiety were also constructed since such a deletion generated functional ICCRs with both the Kir6.2 and Kcv channels. The design of M2_T-09-40 and -09-43 creates a direct contact between the last residue (Cys^457^) of the amphipathic helix VIII of M2 and the TM1 of mTREK-1 (at Val^41^ and Trp^44^, respectively). TEVC recordings showed no significant regulation of M2_T-09-40 and -09-43 by ACh 5 μM (Figures 4A–4C). The control with the G protein-activated potassium channel Kir3.4T demonstrated that, despite its close proximity with TREK-1, the M2 receptor in M2_T-09-40 and -09-43 was still able to activate Gi/o proteins in presence of 5 μM ACh (Figures S4H and S4I). Consequently, the absence of ACh-induced regulation of M2_T-09-40 and -09-43 is not due to a loss of receptor function.

### The regulation of mTREK-1 by the fused M2 receptor is not carried out by intracellular effectors

WT TREK channels can be activated by the Gαi protein-protein kinase A pathway in mammalian cells (Cain et al., 2008; Lesage et al., 2000). To rule out the possibility that the functionality of the M2_T ICCR is not directly due to the fusion of the two partners but related to the activation of the Gαi protein pathway (or other intracellular effectors) and a subsequent indirect effect on the mTREK-1 moiety, four controls were performed. First, the unfused receptor and channel were co-expressed. Figures 4D and 4E shows a slight (−8.9% ± 0.5%) but not significant inhibition in presence of ACh 5 μM (p = 0.0625). Therefore, mTREK-1 is not regulated by the M2 receptor when the two proteins are not fused. This result indicates that the activation of mTREK-1 ICCRs does not rely on intracellular effectors in our experimental conditions (*Xenopus* oocyte, 91 mM extracellular K^+^, Vm = −50 mV, 0.3 mM niflumic acid). The second control is based on the irreversible inactivation of Gi/o proteins by the pertussis toxin (PTX) (Hatcher-Solis et al., 2014). When mRNAs coding for PTX (S1 subunit) and M2_T-00-43 were co-injected, amplitudes of ICCR activation were similar with and without PTX (49.3% ± 4.4% and 49.9% ± 5.1%, respectively; Figures 4D, 4F, and G), indicating that Gi/o proteins are not involved in the activation. Expression and function of PTX were confirmed by the inhibition of Kir3.4T activation by the M2 receptor (Figures 4H–4J). These controls were performed on the same day as the recordings with ICCR. Third, key phosphorylation sites of mTREK-1 (S300 and S333) (Murbartián et al., 2005) were mutated in alanine and the results (Figures S5A–S5D) show an increase of the basal current amplitude as previously reported for these mutants and a preserved activation of the ICCR by ACh. While the activation of the S300A S333A double mutant by ACh is significant, the percentage of ACh-induced activation is lower than the WT, likely due to the high basal activity of the mutated channel or a partial involvement of the G protein-mediated phosphorylation. To conclusively rule out the potential role of G protein pathways in the response of mTREK-1 ICCR, a fourth control was made by inserting the T4L domain in the third intracellular loop of the receptor in M2_T-00-43 in order to completely abolish G protein coupling and activation. The results (Figures 4D and 4K) demonstrate that the G protein-uncoupled ICCR is still activated by ACh with the same amplitude as WT, confirming the G protein-independent regulation of the ICCR.

Altogether, these results demonstrate that the activation of M2_T ICCRs relies on the direct fusion between the two proteins and does not involve indirect intracellular signaling in our experimental conditions.

### The D2 receptor also regulates the fused TREK-1 channel

**Figure 5:**
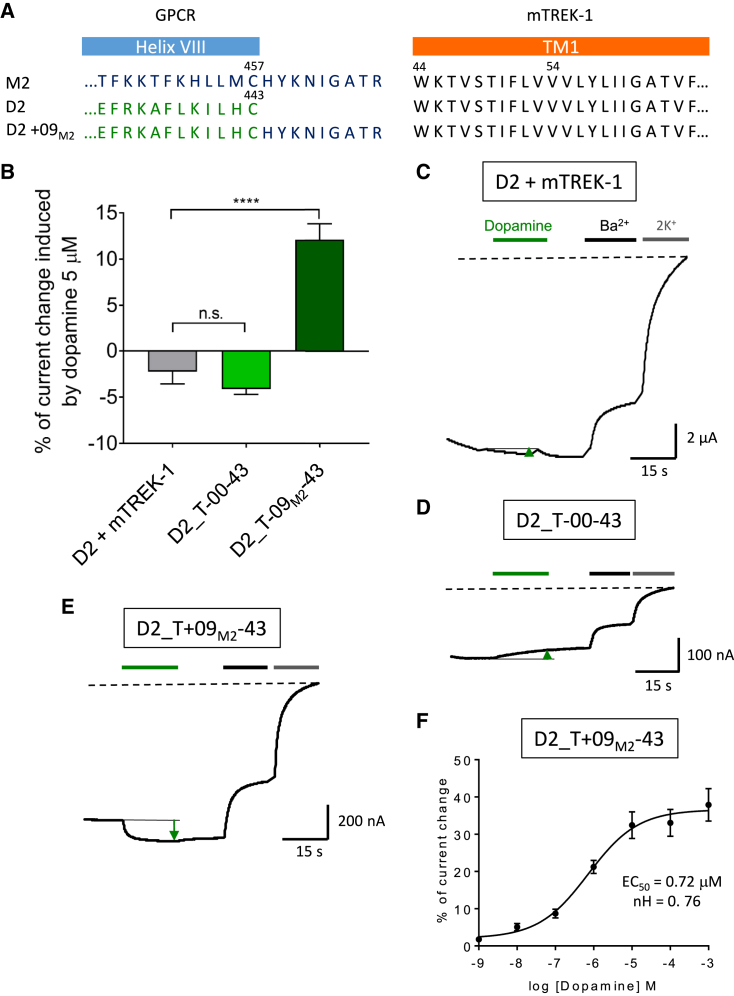
D2_TREK-1 is also ligand regulated with an extended linker length (A) Sequences of GPCR C termini that are fused to the N terminus of mTREK-1, which is truncated of its first 43 residues. In the construct D2_T+9_M2_-43, the C terminus of D2 is extended with the last nine residues of M2 to match the same linker length as M2_T-00-43. (B) Percentage of current change induced by 5 μM dopamine on the unfused D2_L_ and mTREK-1 (D2+mTREK-1, grey bar), the fused D2_T-00-43 (light green bar), and the ICCR with extended D2 C terminus: D2_T+9_M2_-43 (dark green bar). The number of recordings is five, 32, and 17, respectively. One way ANOVA post Dunnett's test; n.s., p = 0.55. ∗∗∗∗p < 0.0001. Error bars are SEM. (C) Representative TEVC recordings of D2 co-expressed with unfused mTREK-1. (D) Representative TEVC recordings of D2_T-00-43. (E) Representative TEVC recordings of D2_T+09_M2_-43. (F) Concentration-effect curve of dopamine on D2_T+9_M2_-43.

To determine whether the regulation of the fused mTREK-1 is not limited to the M2 receptor, as observed with Kcv, we constructed fusions with the D2 receptor. The C terminus of the D2 receptor being nine residue shorter than that of the M2 receptor, the construct D2_T-00-43 connects the last residue of the D2 helix VIII (Cys^443^) to the first helix of mTREK-1 (Trp^44^) (Figure 5A). According to the results obtained with M2_T-09-43, such a connection should not create a functional ICCR. To mimic the linker length of the functional M2_T-00-43 ICCR, the D2 receptor C terminus was extended with the last nine residues from the M2 receptor, yielding a construct named D2_T+09_M2_-43 (Figure 5A). TEVC experiments confirmed that only the latter construct with the extended C terminus (+09_M2_-43) was activated by 5 μM dopamine (12.1% ± 1.7%) while the D2_T-00-43 construct showed a weak inhibition (−4.1% ± 0.6%) that is not significantly different from the control with co-expression of the unfused proteins (−2.2% ± 1.3%) (Figures 5B–5E). The concentration-effect curve on D2_T+09_M2_-43 (Figure 5F) indicates an EC_50_ of dopamine (0.72 μM), which is in the same order of magnitude as the EC_50_ (0.4 μM) measured on the equivalent Kir6.2 ICCR (D2_K+09_M2_-25) (Moreau et al., 2008). The Hill coefficient is 0.76, which is similar to the Hill coefficient observed for ACh on M2_T-00-43. These results demonstrate the ability to build functional TREK-1-based ICCRs with two different GPCRs using a similar design of the linker.

### Fused mTREK-1 preserves its pharmacological regulation and mutants offer additional properties to the signal generated by the ICCRs

**Figure 6:**
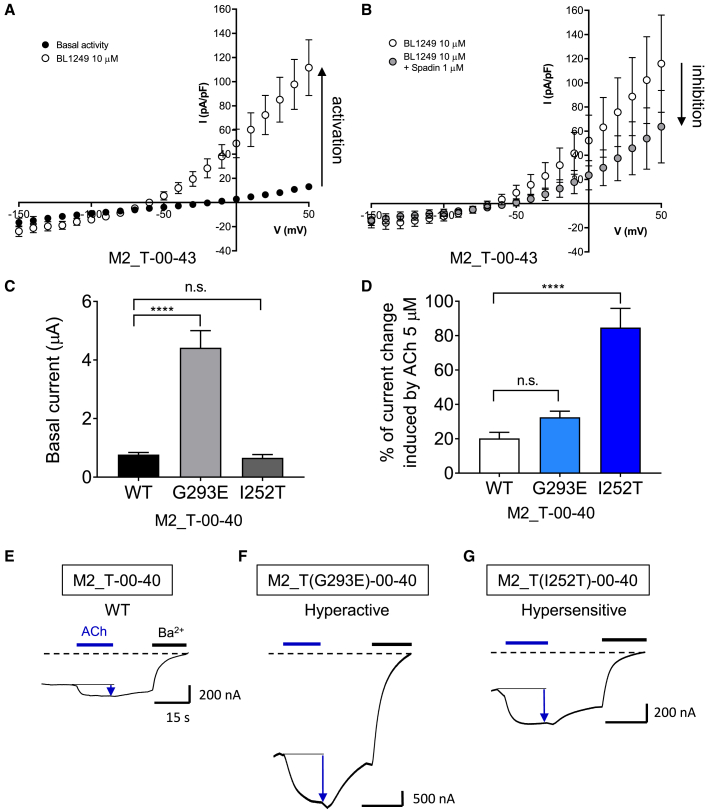
Regulation of mTREK-1 fused to M2 by pharmacological compounds and mutations of the channel induced specific signal properties (A) Current-voltage curves obtained by whole-cell patch-clamp recordings on HEK293 cells expressing M2_T-00-43 and in absence (black dots) or in presence (white dots) of 10 μM activator BL-1249. The black arrow indicates the activation. Error bars are SEM. The number of recordings is 10. (B) Similar experiments performed on another set of cells in presence of 10 μM BL-1249 (white dots) or in presence of 10 μM BL-1249 + 1 μM inhibitor spadin (grey dots). The black arrow indicates the inhibition. The number of recordings is five. Mann-Whitney test: p = 0.03125. (C) Amplitude of the basal current of the indicated mutants of mTREK-1 in M2_T-00-43 expressed in *Xenopus* oocytes and recorded by TEVC. G293E, hyperactive mutant; I252T, hypersensitive mutant. One-way ANOVA post Dunnett's test . n.s., p = 0.95. The number of recordings is 40, 18, and 13 for WT, G293E, and I252T, respectively. (D) Percentage of current change induced by ACh 5 μM on the indicated mutants. One-way ANOVA post Dunnett's test. n.s., p = 0.157. (E) Representative TEVC recordings of M2_T-00-40. (F) Representative TEVC recordings of M2_T(G293E)-00-40. (G) Representative TEVC recordings of M2_T(I252T)-00-43. Error bars are SEM. ∗∗∗∗p < 0.0001.

Adding diversity to the repertoire of ion channels that can be turned in ICCRs would open new possibilities beyond the simplification of the linker design. We checked whether salient pharmacological properties of mTREK-1 were preserved within a fusion protein. To determine whether the fused mTREK-1 was still regulated by pharmacological compounds, the responses of ICCRs to the activator BL1249 (Pope et al., 2018) and to the inhibitor spadin (Mazella et al., 2010) were assessed. M2_T-00-43 was expressed in HEK293 cells by transfection and its functional characterization was performed by whole-cell patch-clamp recordings. The results, depicted in Figures 6A, 6B, S5E, and S5F, show an activation of M2_T-00-43 by 10 μM BL1249 (880% ± 190% increase of the current) and an inhibition of BL1249 activation by 1 μM spadin (56.8% ± 9.1%). These results confirm that the mTREK-1 moiety retains its native pharmacology.

Numerous structure-function studies demonstrated the possibility to tune the properties, or to create specific properties, of ion channels by site-directed mutagenesis (Subramanyam and Colecraft, 2015). In mTREK-1, a new mutation G293E, inspired by the loss-of-function G293R variant inventoried in the SNPs databank COSMIC, yields a hyperactive channel with higher basal activity, while the selectivity filter mutation I252T confers sodium permeability and a hypersensitivity to stretch activation (Decher et al., 2017) (the latter being causal of ventricular tachycardia in humans). Note that our numbering nomenclature is based on the sequence of the isoform mTREK-1a, which is 15 residues shorter than mTREK-1b. Hence, the I252T mutation is equivalent to the I267T mutation studied by Decher et al. (2017).

Both the G293E and I252T mutants of the M2_T-00-40 ICCR were expressed in *Xenopus* oocytes and characterized by TEVC. The results, depicted in Figure 6C, show a higher amplitude of the basal current generated by the mutant G293E (4.42 ± 0.58 μA) compared with WT and with the I252T mutant (0.77 ± 0.07 μA and 0.66 ± 0.11 μA, respectively). Thus, the hyperactive G293E mutant considerably increases (>5-fold) the ICCR signal amplitude, a very desirable property for weakly expressed ICCRs. Application of ACh leads to an activation of the hyperactive ICCR (32.5% ± 3.6%) in a proportion not significantly different from its WT counterpart (20.2% ± 3.5%) (Figures 6D–6F), suggesting that only the basal state of TREK-1 seems affected by the mutation and not the allosteric regulation by the M2 receptor. The mutation I252T affects ion selectivity and increases the stretch sensitivity. In the absence of extracellular Na^+^ and in symmetrical K^+^ concentrations, the basal current of the mutated I252T ICCR was similar to that of the WT (Figure 6C). The activation induced by ACh (84.7% ± 11.2%) was increased more than 4-fold compared with that of the WT counterpart (20.2% ± 3.5%) (Figures 6D, 6E, and 6G). The hypersensitivity of the mutant to stretch stimuli could explain the higher activation of the ICCR by M2. Indeed, the conformation changes of M2 are also mechanically transduced to the mTREK-1 gate(s). While the underlying mechanism remains to be elucidated, such a drastic increase of the ligand-induced responses is of great interest for ICCRs. Altogether, the characteristics of two mTREK-1 mutants are preserved within ICCRs and can be exploited to adjust both the basal and ligand-induced levels of the signal.

## Discussion

The activity of ion channels (gating) and GPCRs (intracellular signaling) is generally regulated by "long-distance" conformational changes coupling the signal reception sites (ligand-binding sites or physical sensors) and the signal generating sites (gates or intracellular effector-activating sites) (Changeux and Christopoulos, 2016). Those structural rearrangements are governed by highly precise molecular mechanisms optimized along the long evolution of living organisms.

Inspired by these natural sensors, numerous studies developed artificial ligand-, light-, or voltage-gated ion channels (Arrigoni et al., 2013; Cosentino et al., 2015; Alberio et al., 2018; Ohndorf and MacKinnon, 2005; Schönrock et al., 2019; Difrancesco et al., 2015; McCoy et al., 2014; Wang and Zocchi, 2011; Coyote-Maestas et al., 2019; Gorman et al., 2019) in order to extend the diversity of recognized ligands or to help understand gating mechanisms or to forge optogenetic tools instrumental in neuroscience. A common challenge in these studies is to artificially create an allosteric transition between two exogenous domains: one for signal recognition and one for signal generation. In the case of ICCR, the concept was initially based on an ion channel (Kir6.2) that naturally evolved toward an allosteric modulation by a large regulatory protein, the sulfonylurea receptor (SUR) (Inagaki et al., 1995). Functional and structural studies highlighted the role of the channel N-terminal domain in SUR-mediated regulation, suggesting that this domain is one of the elements required for the propagation of sulfonylurea-induced conformational changes from SUR toward the channel gate(s). Fusion of GPCRs to this Kir6.2 N-terminal domain indeed created artificial allosteric regulations of Kir6.2 channel, which is related to "discrete, reversible, conformational change in the [receptor] structure" as defined as "allosteric transition" (Changeux and Christopoulos, 2016).

In the case of the virus-encoded Kcv channel, the situation is reversed. Kcv has not evolved toward allosteric regulation, but, on the contrary, it has preserved the most minimalist sequence enabling potassium selectivity and permeation, in order to create a potassium leak that facilitates viral infection. The channel adopts a constitutive open state, which has been observed in several heterologous expression systems (Gazzarrini et al., 2003). Neither the "gating hinge," formed by a glycine involved in transmembrane helix bending required for channel opening, nor bundle-crossing gates in the cytoplasmic side of transmembrane helices are observed in Kcv (Gazzarrini et al., 2003). Despite its minimalist architecture, the artificial regulation of Kcv could be achieved through fusion of exogenous domains. Light- (Cosentino et al., 2015; Alberio et al., 2018), calcium- (Gazzarrini et al., 2003), and voltage-sensitive (Arrigoni et al., 2013) Kcv channels have been engineered by N-terminal fusion of sensor domains. Fusion with the M2 receptor demonstrates that the conformational changes of a transmembrane receptor can also regulate Kcv function via its N-terminal domain. However, the size of the linker differs from those of all previous artificially regulated Kcv channels. Fusion of the D2_L_ dopaminergic receptor to Kcv, with the same linker length, did not generate a functional ICCR. The molecular mechanisms governing the allosteric regulation thus depend both on the fused regulatory domain and on the linker size, and their full understanding would require structural studies revealing the transmission mechanisms from sensors to channels. In terms of engineering, the simplicity of Kcv contrasts with the diversity of allosteric regulations it is amenable to. However, in the absence of a linker transferable from one GPCR to another, Kcv is not the appropriate ion channel for creating large sets of ICCRs with our current knowledge.

The rationale behind the use of Kir6.2 and Kcv channels in ICCRs was based on the existence of modulating subunits for Kir6.2 and earlier protein engineering with soluble sensors for Kcv. By contrast, the second approach, which uses the mTREK-1 channel, was counter-intuitive for three reasons. First, the main natural regulatory domain of mTREK-1, responsible for pressure, temperature, pH, and lipid and fatty acid sensing, lies at the C-terminal end of the transmembrane helices (Soussia et al., 2018; Noël et al., 2011; Pavel et al., 2020), while we have designed N-terminal fusions. Second, mTREK-1 is devoid of an interfacial helix at the N-terminal domain, which is deemed instrumental for the efficient GPCR to channel coupling in Kir6.2 and Kcv. Third, the channel is dimeric, not tetrameric like most other potassium channels, implying a different regulation of the gating, and possibly precluding the ability of the fused GPCRs to contact each other. However, attaching GPCRs to the truncated N terminus of mTREK-1 created positive allosteric regulation of the channel when the first 40, 43, or 46 residues were truncated, the 43 being optimal. Attempts to reduce the linker size and directly connect the GPCR helix VIII to the mTREK-1 TM1 (M2_T-09-XX) abolished the regulation, in contrast with Kir6.2- and Kcv-based ICCRs.

The straightforward transfer of design to constructs with the dopaminergic D2 receptor confirmed that mTREK-1 can be fused and regulated by different GPCRs, provided that their C terminus extends nine residues beyond the palmitoylated cysteine of the helix VIII. These results demonstrate the potential of mTREK-1 to create functional ICCRs with different GPCRs, using a simplified and consensus strategy. This ion channel is consequently a promising candidate for designing a new generation of ICCRs. Moreover, mutations of mTREK-1 expand the properties of the ICCR signal by increasing ∼6-fold the amplitude of the basal current and ∼4-fold the amplitude of the ligand-induced response with the mutants G293E and I252T, respectively.

Another particularity of mTREK-1 is its oligomeric state. While Kir6.2 and Kcv ICCRs contain four receptors per pore, mTREK-1 ICCRs impose a new stoichiometry of two GPCRs per pore and demonstrates that two receptors on two opposite sides of the pore are sufficient to open the channel. The Hill coefficients for ACh and CCh were under 1 for both the tetrameric Kcv and dimeric mTREK-1 ICCR, indicating that no cooperativity occurs in the regulation of the fused channels by M2. The imposed oligomeric states of the GPCR could be used as a new approach to evaluate the role of receptor oligomerization in other GPCR-based mechanisms. Moreover, an increasing number of studies demonstrate the physiological role of K_2P_ channels heterodimerization (Levitz et al., 2016; Blin et al., 2016). In the absence of reliable tools (e.g., specific antibodies, direct assays) for studying specific K_2P_ channels, ICCRs could be an alternative method.

In perspective, the similarity of structure between different K_2P_ (TWIK-1, Miller and Long, 2012; TASK-1, Rödström et al., 2020; TASK-2, Li et al., 2020; and TRAAK, Brohawn et al., 2012, 2013, 2014) suggests that the ICCR technology could be effectively extrapolated to other members of the K_2P_ family with the objectives of (1) creating pharmacological regulation based on the large repertoire of GPCR ligands to compensate the paucity of known pharmacological compounds acting on these K_2P_ channels, (2) broadening the properties of ICCR signals, or (3) studying the gating regulation of specific members of the family.

An additional benefit offered by the diversity of ion channels is the ability to modify the properties of ICCRs for increasing the signal amplitude for adding sensitivities to different stimuli (e.g., ligands, temperature, stretch) or for blocking the signal with specific chemicals or toxins. mTREK-1 has the distinction of being regulated by a large diversity of stimuli (pH, mechanical activation, temperature, volatile anesthetics, poly-unsaturated fatty acids, and phosphorylation), which offers a large choice of tests to control the presence and function of ICCRs in cells or artificial environments such as biosensing platforms.

This study demonstrates that various potassium channels from different families can be allosterically regulated via their N-terminal fusion with a GPCR. This fusion strategy could be extended to a broader range of ion channels with a cytoplasmic N terminus, even if this domain has no known role in gating regulation. In consequence, these results open new horizons for the generation of various man-made LGICs with tunable electrical signal, stimuli, or GPCR stoichiometries.

### Limitation of the study

The tested receptors belong to the class A (rhodopsin-like) GPCRs. Fusing GPCRs from others classes could generate different results. In particular, the fusion of mTREK-1 to class C GPCRs should disrupt the formation of mandatory dimers.

In most cases, the C terminus length of the GPCR must be adjusted, which could affect interactions with cognate proteins, internalization, recycling, and phosphorylation of the fused and engineered receptor.

The surface expression level of the ICCR is dependent on the fused receptor, which could be a limiting factor for measuring significant current amplitude.

## STAR★Methods

### Key resources table

**Table:** 

REAGENT or RESOURCE	SOURCE	IDENTIFIER
**Biological samples**		

Lab Bred Adult *Xenopus laevis* Female Frog	Centre de ressources biologique, Rennes, France	RRID:XEP_Xla

**Chemicals, peptides, and recombinant proteins**		

Acetylcholine chloride	Sigma-Aldrich	Cat# A6625
Atropine	Sigma-Aldrich	Cat# A0132
BL-1249	Sigma-Aldrich	Cat# B2186
Dopamine hydrochloride	Sigma-Aldrich	Cat# H8502
Oxytocin	Vadim Cherezov’s laboratory	N/A
Spadin	Catherine Heurteaux’s laboratory	N/A
jetPEI®	PolyPlus Transfection	Cat# 101-10N
Collagenase	Sigma-Aldrich	Cat# C9891
Penicillin/Streptomycin	Sigma-Aldrich	Cat# P0781
Gentamycin sulfate	Sigma-Aldrich	Cat# G1264
Niflumic acid	Sigma-Aldrich	Cat# N0630

**Critical commercial assays**		

QIAfilter Plasmid Midi Kit	Qiagen	Cat# 12245
mMESSAGE mMACHINE™ T7 ULTRA Transcription Kit	Thermo Fisher Scientific	Cat# AM1345
QuikChange Site-Directed Mutagenesis Kit	Agilent	Cat# 200519

**Deposited data**		

Analyzed data		

**Experimental models: Cell lines**		

HEK293	Invitrogen	Cat# R705-07

**Recombinant DNA**		

Plasmid pGEMHE-derived	Moreau et al., 2015	https://doi.org/10.1016/bs.mie.2014.12.017
Plasmid pXOOM	Jespersen et al., 2002	https://doi.org/10.2144/02323st05
Plasmid pIRES2-eGFP	Clontech	Cat# 6029-1
M2 pGH2	Moreau et al., 2008	https://doi.org/10.1038/nnano.2008.242
D2 pGH2	Moreau et al., 2008	https://doi.org/10.1038/nnano.2008.242
OXTR[T4L]	Niescierowicz et al., 2014	https://doi.org/10.1016/j.str.2013.10.002
mTREK-1 pEXO	Fink et al., 1996	https://doi.org/10.1002/j.1460-2075.1996.tb01077.x
Kcv (BLINK2 pDONR)	Alberio et al., 2018	https://doi.org/10.1038/s41592-018-0186-9
Kir3.4T pXOOM	Vivaudou et al., 2017	https://doi.org/10.1007/978-1-4939-7151-0_15
PTX-S1	Vivaudou et al., 1997	https://doi.org/10.1074/jbc.272.50.31553

**Software and algorithms**		

eeFit	Vivaudou, 2019	https://doi.org/10.2144/btn-2018-0136
GraphPad Prism 7	GraphPad software	https://www.graphpad.com/scientific-software/prism/
Clampex 9	Axon Instrument	https://moleculardevices.app.box.com/s/d93nukl3chbo206t33cw5fpabsph6wh4

**Other**		

HiClamp	MultiChannel Systems	https://www.multichannelsystems.com/products/hiclamp
MultiClamp 700A patch clamp amplifier	Molecular Devices	https://www.moleculardevices.com/products/axon-patch-clamp-system/amplifiers/axon-instruments-patch-clamp-amplifiers
12-bit analog-to-digital converter Digidata-1322A	Axon Instrument	https://www.moleculardevices.com/products/axon-patch-clamp-system/digitizers/axon-digidata-1550b-plus-humsilencer

### Resource availability

#### Lead contact

Further information and requests for resources and reagents should be directed to and will be fulfilled by the lead contact, Christophe Moreau (christophe.moreau@ibs.fr).

#### Materials availability

All the materials used in this study are commercially available. Plasmids generated in this study are available from the lead contact upon request.

### Experimental model and subject details

#### Cell culture and transfection

HEK293 cells were grown in 75-mm tissue-culture dishes (Falcon, Franklin Lakes, NJ) in Dulbecco’s modified Eagle’s medium (Gibco, Life Technologies, Saint Aubin, France) supplemented with 10% fetal calf serum (Hyclone, Thermo Fisher Scientific GMBH, Ulm, Germany) and 1% penicillin/streptomycin (Gibco, Life Technologies, Saint Aubin, France) in a humidified incubator at 37 °C (5% CO_2_). For electrophysiology of ICCRs, 0.8 mg of plasmid was transfected using Jet-PEI (PolyPlus Transfection, Illkirch FRANCE) according to the manufacturer’s instructions. Cells were plated onto 35-mm dishes 24 h before transfection, and experiments were performed over the following 1–2 days.

#### Xenopus Oocytes and RNA Microinjection

Xenopus oocytes were prepared as previously reported (Moreau et al., 2008). Briefly, after surgical retrieval, the oocytes were defolliculated by type 1A collagenase. Each oocyte was injected with 50 nl of RNAse-free water containing the desired mRNAs. The mRNA quantities used per oocyte were: ICCR with Kcv, 2.7 ng (M2_Kcv and D2_Kcv) and 2.9 ng (OXTR_Kcv); ICCR with mTREK-1, 4.2 ng; ICCR with Kir6.2, 4.3 ng; M2, 2.3 ng; D2, 2.2 ng; Kir3.4T, 2.2 ng; Kcv 0.46 ng and mTREK-1, 2.1 ng. Microinjected oocytes were incubated 2 to 3 days at 19°C in modified-Barth's solution (in mM: 1 KCl, 0.82 MgSO_4_, 88 NaCl, 2.4 NaHCO_3_, 0.41 CaCl_2_, 16 HEPES, pH 7.4) supplemented with 100 U.ml^−1^ of penicillin and streptomycin and 0.1 mg.mL^−1^ of gentamycin. Animal handling and experiments fully conformed to European regulations and were approved by the French Ministry of Higher Education and Research (APAFIS#4420-2016030813053199 v4 to CM). Authorization of the animal facility has been delivered by the Prefect of Isere (Authorization #D 38 185 10 001).

### Method details

#### Molecular biology

The mTREK-1 isoform used in this study is mTREK-1a (Uniprot #P97438) and numbers of residues are defined accordingly. The genes coding for ICCRs, the unfused M2, Kcv and mTREK-1 were inserted in pGEMHE-derived vectors which contains globin untranslated regions for enhancing protein expression in Xenopus oocytes. Unfused Kir3.4T gene was inserted in pXOOM vector which has been designed for dual protein expression in Xenopus oocytes and in mammalian cells (Jespersen et al., 2002). Except for constructs M2_Kcv-09-06 to -09-12, D2_Kcv-00-03, OXTR[T4L]_Kcv-42-03 (GenScript), M2_T-09-43 and D2_T-00-43 (ATG:biosynthetics), M2_T-00-46, M2[T4L]_T-00-43, M2_T-00-43 mutated in the phosphorylation sites of the channel (S300A and S333A) and the N-terminal truncated mTREK-1 (GeneCust), that were synthesized, ICCRs with Kcv and mTREK-1 channels were created by two steps PCRs with the QuikChange site-directed mutagenesis kit (Agilent). The first PCR aims to amplify the gene of the ion channels with flanking regions that hybridize in 5′ with the GPCR 3′ sequence and in 3′ with the plasmid sequence at the site of insertion. Positive clones were verified by sequencing. DNA was amplified using Qiagen MidiPrep Kit. After cDNA linearization in the 3′ end of a polyA tail, mRNA was synthesized using the mMessage mMachine T7 Ultra Kit (Thermo Fisher Scientific) and purified by the standard phenol:chloroform protocol, analyzed by agarose-gel electrophoresis and quantified by spectrophotometry (Moreau et al., 2017). For the experiments with HEK cells the ICCRs were cloned into pIRES2-eGFP vector (Clontech) and the sequence verified by DNA sequencing.

#### Electrophysiological recordings in HEK293 cells

Pipettes were pulled from hematocrit-capillaries (Hirschmann Laborgeraete, Germany) using a vertical puller (PC-10, Narishige International, London, United Kingdom), and had resistances of 3–6 MΩ when filled with internal solution and measured in standard bath solution. Whole cell membrane currents were measured and filtered at 3 kHz by a MultiClamp 700A patch clamp amplifier (Molecular Devices), and digitized at 10 kHz using a 12-bit analog-to-digital converter Digidata-1322A (Axon Instrument, Sunnyvale, CA, United States). Recordings were done using Clampex 9 software (Axon Instrument). The external solution used to record mTREK-1 currents in HEK293 cells contained (in mM): 140 NaCl, 5 KCl, 3 MgCl_2_, 1 CaCl_2_, 10 HEPES, pH 7.4, with NaOH. The intra-pipette solution contained (in mM): 145 KCl, 3 MgCl_2_, 5 EGTA, 10 HEPES, pH 7.2, with KOH. mTREK-1 activator BL1249 was dissolved at a concentration of 10 μM and spadin at 1 μM in external solution. Experiments were performed at room temperature.

#### Electrophysiological recordings in xenopus oocytes

Two-electrode voltage-clamp (TEVC) recordings were performed automatically with HiClamp robots (MultiChannel Systems). These TEVC robots rapidly change the applied solutions by transferring the oocytes in different wells of a 96-well plate. The kinetics of traces is therefore faster than previous studies that used a manual perfusion system. Microelectrodes were filled with 3 mM KCl. During recordings, oocytes were incubated in low potassium concentration buffer (ND96, in mM: 96 NaCl, 2 KCl, 1.8 CaCl_2_, 1 MgCl_2_, 5 HEPES, pH 7.4 supplemented with 0.3 niflumic acid), and in high potassium buffer (in mM: 91 KCl, 1.8 CaCl_2_, 1 MgCl_2_, 5 HEPES, 0.3 niflumic acid, pH 7.4). The membrane voltage was clamped to −50 mV. Ligands and BaCl_2_ were diluted in the high potassium buffer. Ba^2+^ (3 mM) was used as a generic potassium channel blocker with a partial blocking on mTREK-1. Ligand effects were measured in high potassium concentration buffer with the membrane potential clamped to −50 mV and at 19°C.

Change of potassium current induced by ligand was calculated either with the barium-sensitive currents as a 0-reference for Kcv-based ICCRs or with the currents in ND96 for mTREK-1 ICCRs. Softwares created by Michel Vivaudou (Vivaudou, 2019) were used to extract the raw data.

### Quantification and statistical analysis

The statistical analyses were performed with GraphPad Prism 7 (GraphPad Software). All values are represented as mean ± SEM. The number of biological replicates can be found in relevant figure legends. D'Agostino & Pearson normality test was performed before the significance tests. For normal distributed data, statistical tests were done with one-sample t test, unpaired t test or one-way ANOVA post Dunnestt's test, when appropriate. For non-normal distributed data, Mann-Whitney test or Kruskal Wallis test post Dunn's test were used, when appropriate. Statistical significance was defined as p < 0.05, not significant was indicated as "n.s.". The statistical details can be found in the relevant figure legends.

## Data Availability

All data reported in this paper will be shared by the lead contact upon request. This paper does not report original code. Any additional information required to reanalyze the data reported in this paper is available from the lead contact upon request.

## Supplemental information

Document S1. Figures S1–S5

Document S2. Article plus supplemental information
