# Supplementary material for: Distinct classes of potassium channels fused to GPCRs as electrical signaling biosensors
Source: Cell Rep Methods. 2021 Nov 22;1(8):100119. doi: 10.1016/j.crmeth.2021.100119 (PMC8688152; doi:10.1016/j.crmeth.2021.100119)
Supplement: Document S1. Figures S1–S5 [file mmc1.pdf]

Cell Reports Methods, Volume *1*

Supplemental information

Distinct classes of potassium channels fused to GPCRs as electrical signaling biosensors

M. Dolores García-Fernández, Franck C. Chatelain, Hugues Nury, Anna Moroni, and Christophe J. Moreau

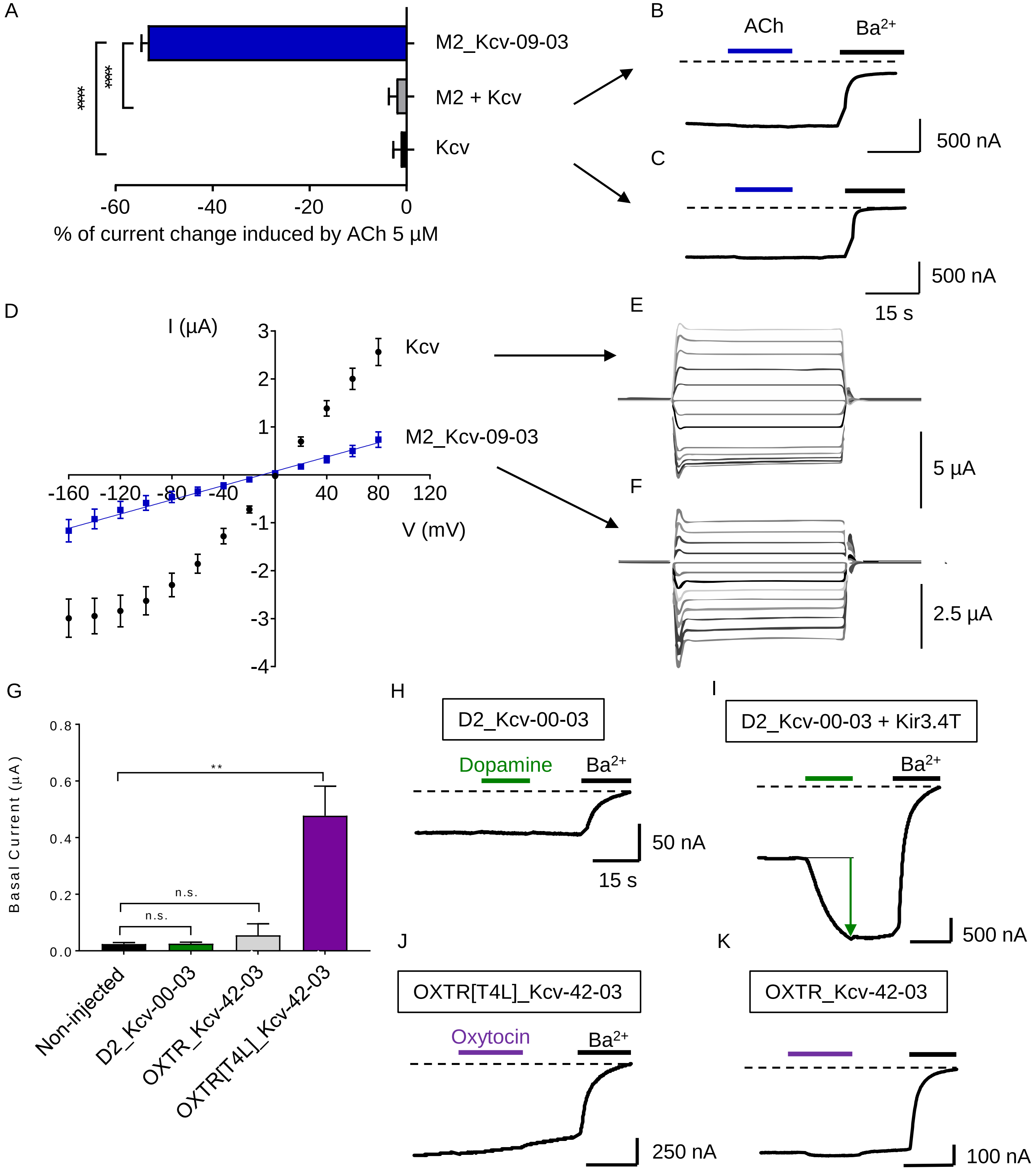

<u>Figure S1: Control of independence to intracellular pathways and voltage dependency of M2_Kcv ICCR and extrapolation to other GPCRs, Related to figure 1.</u>

A-C) Negative controls of acetylcholine (ACh) regulation with unfused M2 and Kcv, and with Kcv alone. Unpaired t test; **** < 0.0001. **D-F**) I/V curves and of basal currents in steady-state of Kcv (black dots) and M2_Kcv-09-03 (blue dots) and representative TEVC recordings of 500-ms sweeps with 20mV increment from -180 to +80 mV with a holding potential of -20 mV. **G-K**) Basal current and representative recordings of the indicated constructs. Unpaired t test; n.s.: P=0.876 and P=0.544 for D2_Kcv-00-03 and OXTR_Kcv-42-03, respectively. **: P=0.0086. Error bars are SEM.

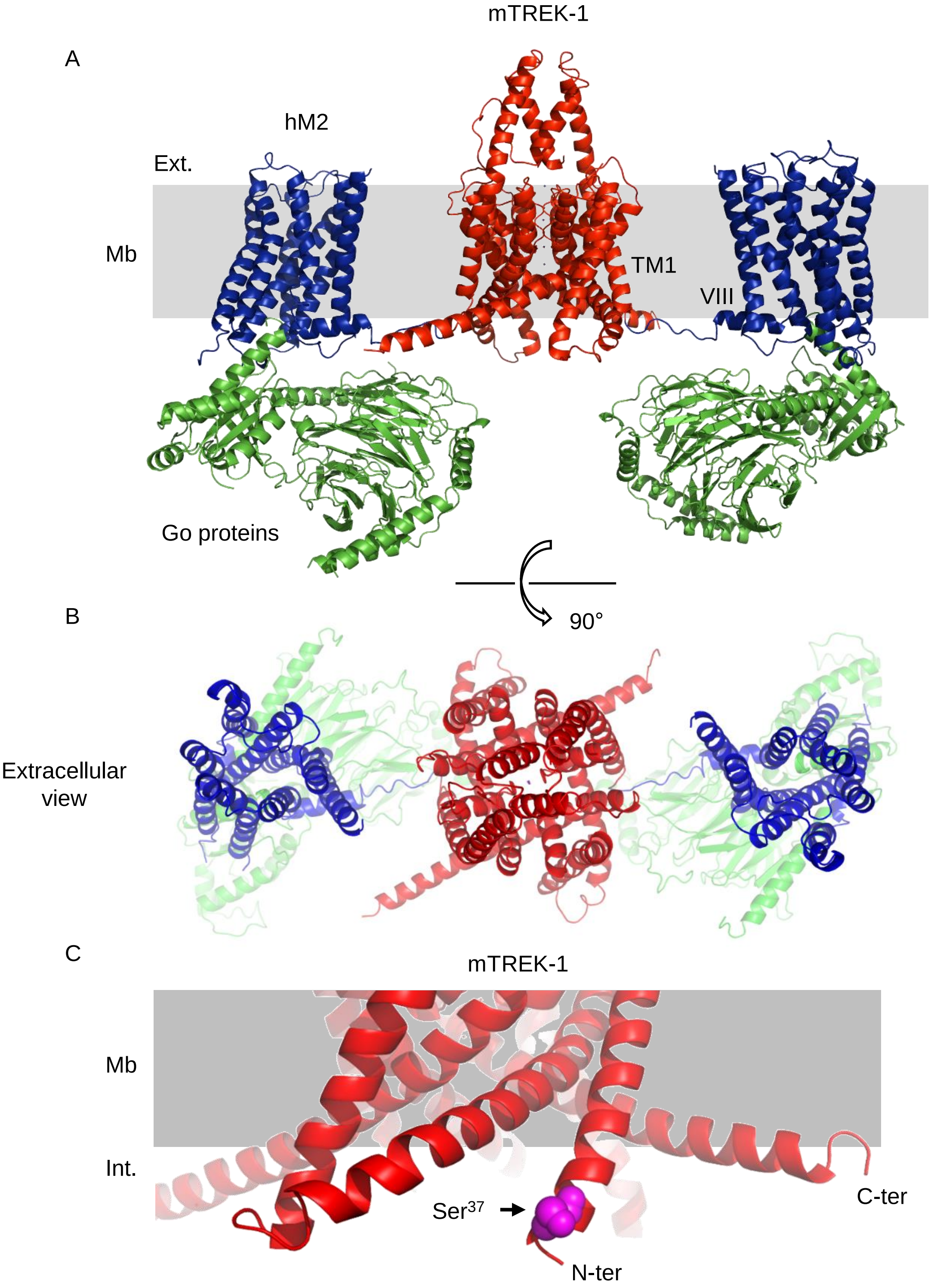

Figure S2: Stoichiometry and structural insight into the fusion of mTREK-1 with M2 receptor, Related to figure 3. **A-B**) Diagram of hM2_mTREK-1-00-43 based on the structure of the human muscarinic M2 receptor (Maeda et al., 2019) (PDB code: 6OIK) with Go proteins in green and the structure of mTREK-1 ($K_{2P}2.1$) (Lolicato et al., 2017) (PDB code: 6CQ6). **C**) Focus on the N-terminus of mTREK-1 (PDB code: 6CQ6) showing the Serine 37 (Ser^{37}, pink), which is replaced by the last residues of M2 in M2_T-00-37.

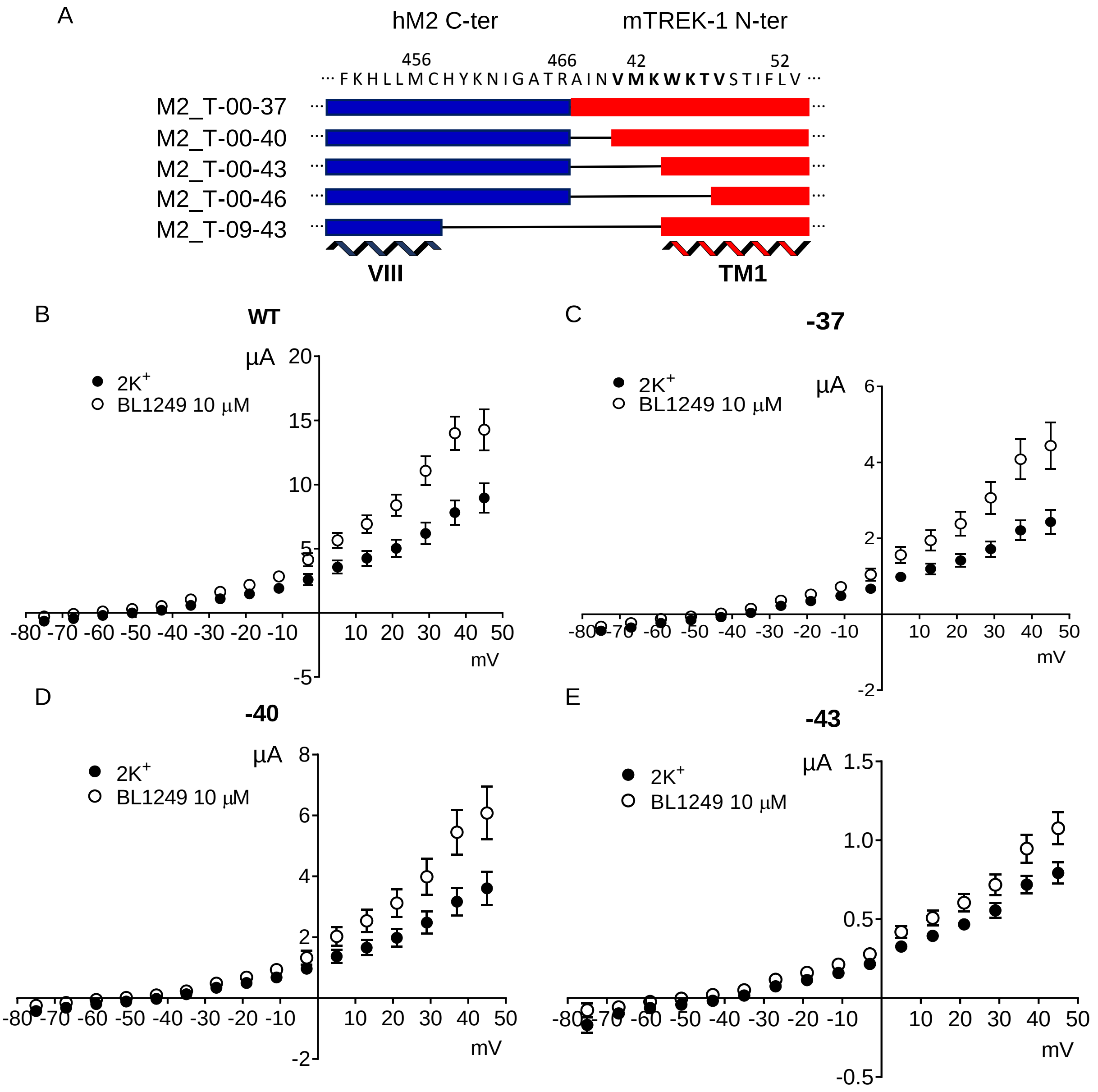

<u>Figure S3: Design of M2_T fusions and functional characterization of N-terminally truncated mTREK-1 channels, Related to figure 3.</u>

A) Alignment of the linking region between hM2 C-terminus (blue) and mTREK-1 N-terminus (red). The sequence in bold is a conserved region in the TREK channels family. **B-E**) I/V curves of the mTREK-1 channels without (B) or with N truncations of the first 37 residues (C), 40 residues (D) or 43 residues (E). Basal currents in low potassium buffer ($2K^+$) are shown in black dots and BL1249-induced currents in white dots. Values are mean ± SEM.

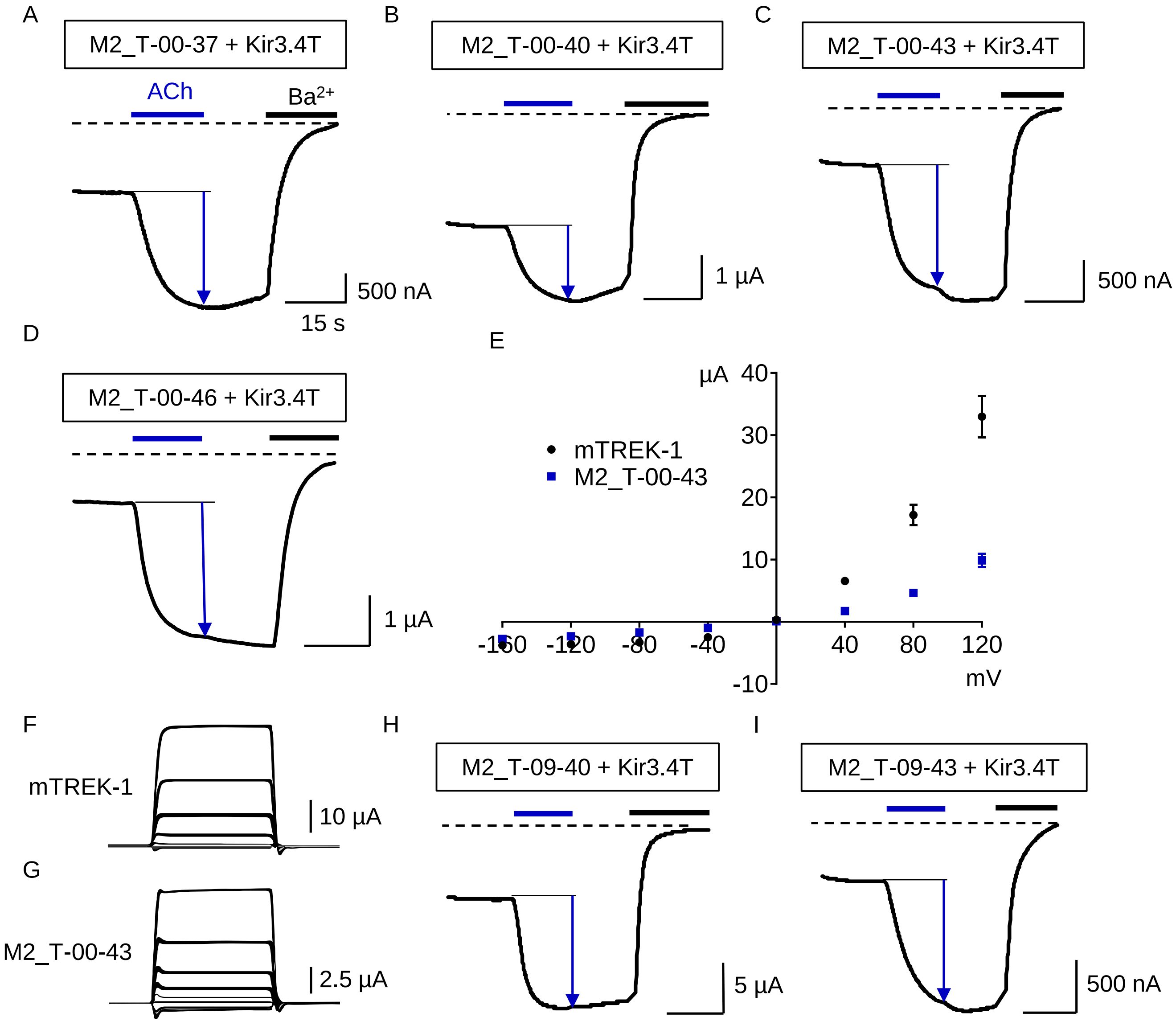

Figure S4: G protein activation assay and voltage-dependence of M2_T ICCRs, Related to figures 3&4.

A-D) Representative TEVC recordings of oocytes co-expressing the G protein-activated Kir3.4T channel with the indicated hM2_mTREK-1 ICCRs. ACh was applied at 5 µM and the blue arrow indicates the G protein-induced activation of the Kir3.4T channel. **E**) I/V in steady-state and in symmetrical potassium buffer of mTREK-1 alone (black dots) or M2_T-00-43 (blue dots). **F-G**) Representative TEVC recordings in basal state of the indicated constructs of 500-ms sweeps with 40 mV increment from -160 to +120 mV with a holding potential of -80 mV. **H-I**) Representative TEVC recordings of G protein-activated Kir3.4T channels by the indicated M2_T constructs with M2 C-terminus truncated of 9 residues (-09). The blue arrows show an activation of the Kir3.4T channel in presence of 5 µM ACh.

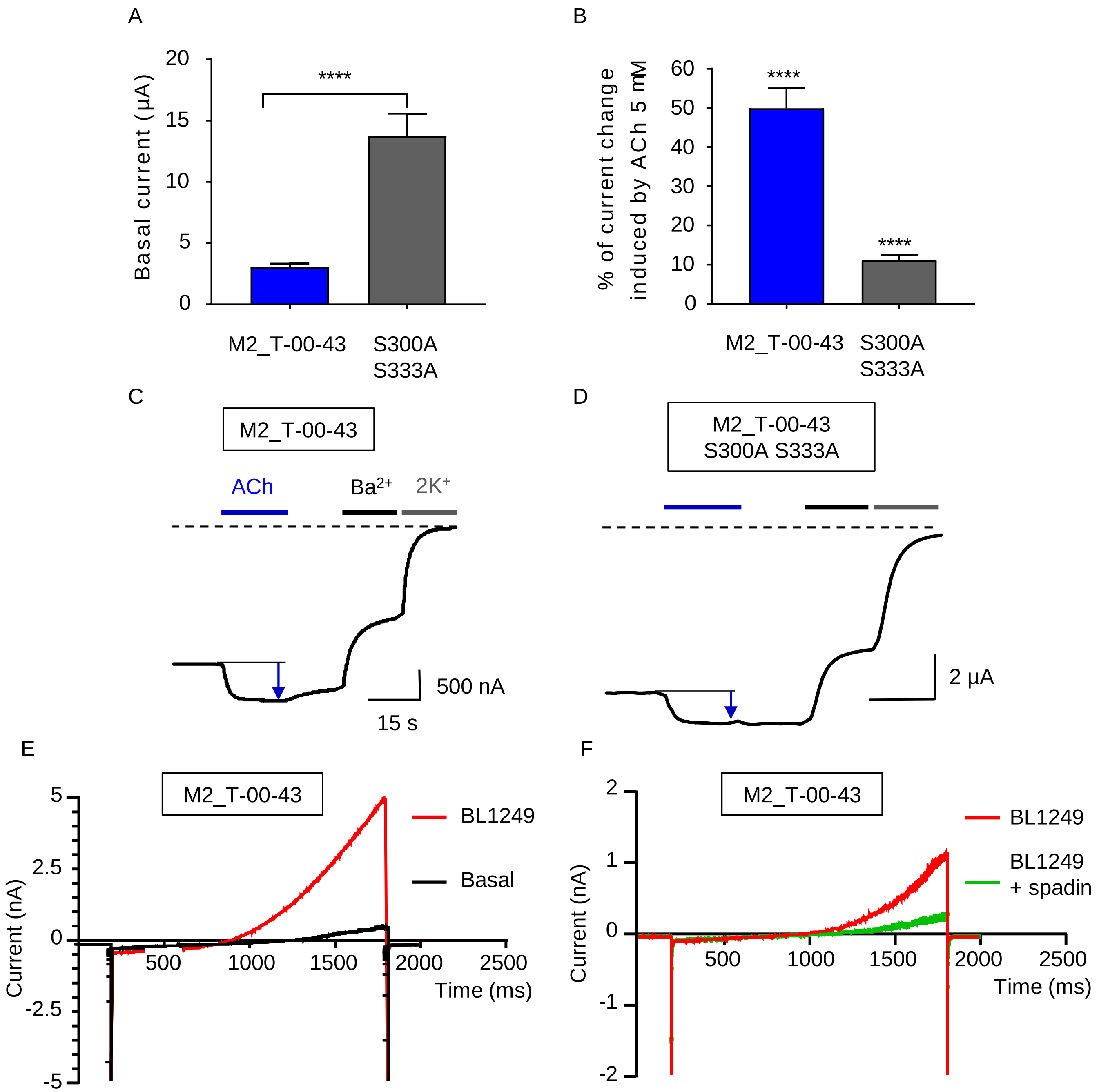

Figure S5: Charactetization of phosphorylation-deficient double mutant and pharmacological characterization of M2_T-00-43, Related to figures 4&6.

A) Basal currents of M2_T-00-43 (blue bar) and the double mutant S300A and S333A (grey bar). One sample t test. **B**) Percentage of current change induced by ACh 5µM on the indicated constructs. Mann Whitney test (ref. = 0). **C-D**) Representative TEVC recordings of (B). **E-F**) Whole-cell patch-clamp traces on HEK293 cells transiently expressing M2_T-00-43 and representative of the Fig. 6A&B. Basal currents are shown in black, 10 µM BL1249-induced currents (activation) in red and co-application of 10 µM BL1249 and 1µM spadin (inhibition) in green. **** $P<0.0001$.
